# Gut Permeability and Microbiota in Parkinson’s Disease: Mechanistic Insights and Experimental Therapeutic Strategies

**DOI:** 10.3390/ijms26199593

**Published:** 2025-10-01

**Authors:** Yicheng Liang, Yuhang Zhao, Alessio Fasano, Chien-Wen Su

**Affiliations:** 1 College of Biological Science, University of California-Davis, One Shields Avenue, Davis, CA 95616, USA; danliang@ucdavis.edu; 2 Mucosal Immunology and Biology Research Center, Massachusetts General Hospital, Harvard Medical School, Charlestown, MA 02129, USA; afasano@mgh.harvard.edu; 3 Department of Neurology, Massachusetts General Hospital, Harvard Medical School, Charlestown, MA 02129, USA; yzhao46@mgh.harvard.edu

**Keywords:** Parkinson’s disease (PD), gut permeability, leaky gut, neuroinflammation, gut microbiota, therapeutic approaches, personalized therapy

## Abstract

Globally, Parkinson’s disease (PD) is the neurodegenerative condition with the most rapidly increasing prevalence, and a growing body of evidence associates its pathology with impairments in the gut–brain axis. Traditionally viewed as a disease marked by the loss of dopaminergic neurons, emerging evidence emphasizes that chronic neuroinflammation is a driver of neurodegeneration, with gut-originating inflammation playing a crucial role. Increased intestinal permeability, often called “leaky gut,” allows harmful substances, toxins, and misfolded α-synuclein into the systemic circulation, potentially exacerbating neuroinflammation and spreading α-synuclein pathology to the brain through the vagus nerve or compromised blood–brain barrier (BBB). This review synthesizes current insights into the relationship between gut health and PD, emphasizing the importance of gut permeability in disrupting intestinal barrier function. This paper highlights innovative therapeutic approaches, particularly personalized therapies involving gut microbiome engineering, as promising strategies for restoring gut integrity and improving neurological outcomes. Modulating specific gut bacteria to enhance the synthesis of certain metabolites, notably short-chain fatty acids (SCFAs), represents a promising strategy for reducing inflammatory responses and decelerating neurodegeneration in Parkinson’s disease.

## 1. Introduction

**Figure 1:**
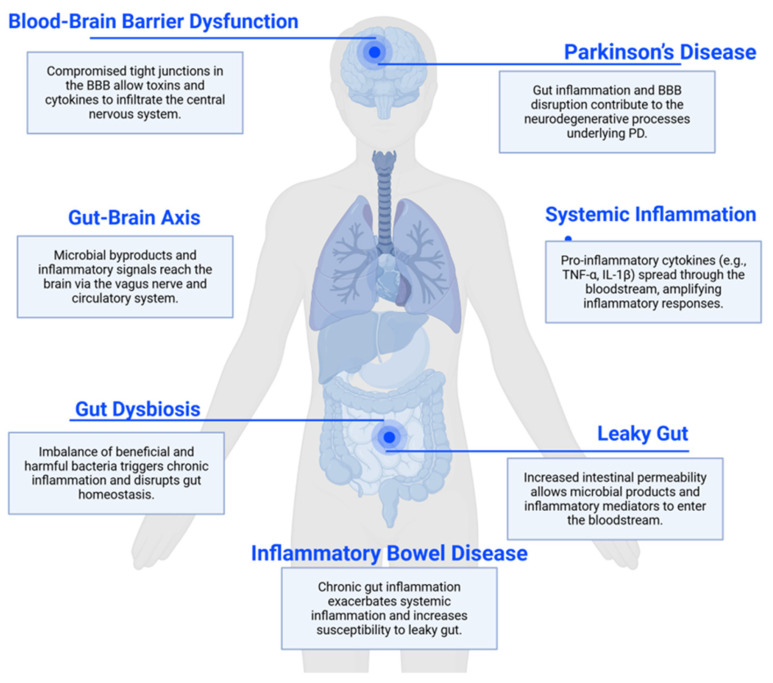
Connection between leaky gut and Parkinson’s disease through the gut–brain axis. This image was created with BioRender.com (accessed on 20 July 2025). Parkinson’s disease (PD); blood–brain barrier (BBB); Tumor Necrosis Factor-Alpha (TNF-α); Interleukin-1 beta (IL-1β).

As a prevalent neurodegenerative disorder, Parkinson’s disease (PD) predominantly occurs in people over sixty years of age, with roughly one percent of this population affected. Throughout North American populations, the disorder appears in about 572 individuals per 100,000, although specific rates differ based on regional and demographic characteristics [1]. Emerging evidence indicates that PD involves a more complex pathophysiology than previously understood, which includes roles for intestinal permeability and the gut microbiome, moving beyond its traditional characterization as a disorder of motor symptoms caused by dopaminergic neuron loss. The association between intestinal disorders and PD has long been established. Growing clinical data support a bidirectional relationship between gastrointestinal (GI) inflammation and neurodegeneration, aligning with the concept of the ‘gut–brain axis’. There are two main models of the origin of PD, the brain-first and body-first hypothesis, according to the supposed initiation of α-synuclein aggregation. Braak’s hypothesis, as the representative body-first hypothesis observed α-syn aggregation in the stomach and lower esophagus of patients with PD but not in those without PD [2]. Supporting this, animal studies have shown that duodenal and pyloric injected α-syn preformed fibrils (PFF) can travel to the brain via the vagus nerve, leading to symptoms like PD, such as motor issues and cognitive decline. The spread of α-synucleinopathy from the gut to the brain can be severed by truncal vagotomy [3]. Vagotomy reduces but does not completely eliminate PD-like pathology, suggesting other contributing mechanisms or pathways. PD patients exhibit non-motor symptoms (e.g., constipation, depression, cognitive decline) that may arise from peripheral pathology (e.g., Enteric Nervous System dysfunction) or brain regions unaffected by vagal spread [4]. Increased intestinal permeability (“leaky gut”) plays a critical role by allowing toxins and pathogens to enter the bloodstream, which may exacerbate neurodegenerative processes. A recent study supports the hypothesis that PD originates in the gut. Analysis of 9350 patients undergoing upper GI endoscopic biopsy revealed a 76% increased risk of PD associated with GI mucosal damage, including erosion, rupture, or ulceration. Notably, GI symptoms often precede motor symptoms and PD diagnosis by over a decade [5], with recent evidence linking upper GI mucosal damage to an increased long-term risk of PD, underscoring the gut’s potential role in prodromal disease. Further recent reviews have expanded on this idea, emphasizing that the gut–brain axis and/or microbiota–gut–brain axis—including microbial metabolites, immune activation, and barrier impairment—may be key drivers of initiating and perpetuating neurodegenerative processes, including PD [6] (Figure 1) Paracellular leakage is a direct consequence of impaired tight junction function. Key players in this dysfunction include transmembrane proteins (e.g., claudins, occludins) and their cytoplasmic partners (e.g., ZO-1/ZO-2), whose disruption leads to intestinal hyperpermeability [7]. This condition is influenced by both genetic factors and environmental elements, which include diet and toxins [8,9]. Various factors contribute to increased intestinal permeability. An unhealthy diet, especially pro-inflammatory substances like refined carbohydrates, gluten, added sugars, can greatly elevate the risk of leaky gut [10]. Additionally, environmental factors that may influence gut health include external stressors such as additives, preservatives, heavy metals, and other chemicals and toxins, along with chronic stressors such as sleep disorders, smoking, and alcohol abuse [11,12].

A healthy intestinal barrier is essential for preventing systemic inflammation, which is linked to various health issues, including PD [13]. The importance of personalized therapy, particularly through gut microbiome engineering and stem cell transplantation, is increasingly being recognized as a promising strategy for establishing gut microbial homeostasis, restoring gut integrity, and enhancing neurological health. In parallel, induced pluripotent stem cell-based therapies (iPSCs) are gaining traction for their dual potential to replace lost dopaminergic neurons and model patient-specific disease mechanisms, offering another avenue for personalized intervention in PD [14,15]. Individualized interventions targeting the gut microbiome may alleviate inflammation and improve neurological outcomes. This review aims to synthesize current insights into the relationship between gut health, blood–brain barrier (BBB) permeability, and PD, highlighting how compromised barriers contribute to neurodegeneration and emphasizing the need for innovative, personalized treatment strategies to improve patient outcomes and quality of life (Figure 1).

## 2. Intestinal Barrier and Gut Permeability

**Figure 2:**
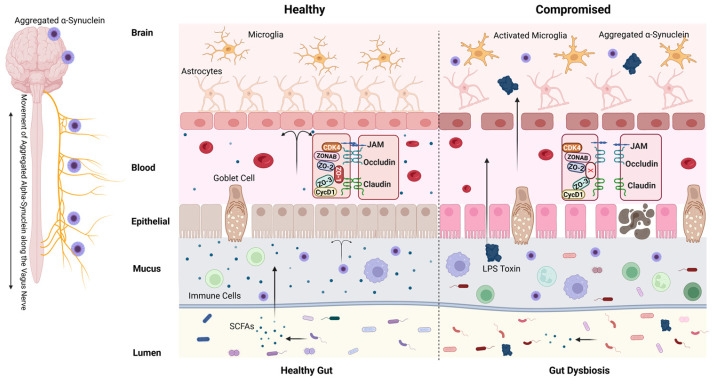
Proposed mechanism linking gut permeability to Parkinson’s disease. Disruption of intestinal tight junctions increases gut permeability, allowing bacterial products (e.g., LPS) and dietary antigens to enter the bloodstream. This promotes systemic inflammation, activates immune cells, and leads to a compromise of the BBB. In parallel, aggregated α-synuclein originating in the gut may travel to the brain via the vagus nerve, seeding neurodegenerative processes. Within the brain, systemic inflammation contributes to microglial activation, further α-synuclein aggregation, and dopaminergic neurodegeneration. Zonulin-mediated tight junction disassembly and dysbiosis-induced reduction in SCFAs exacerbate barrier dysfunction. Potential therapeutic targets include zonulin antagonists and interventions that modulate the microbiome. This image was created with BioRender.com (accessed on 20 July 2025). Lipopolysaccharide (LPS); short-chain fatty acids (SCFAs); junctional adhesion molecule (JAM); Cyclin-Dependent Kinase 4 (CDK4); Y-box binding protein 3 (ZONAB); Tight junction protein 1 (ZO-1); Tight junction protein 2 (ZO-2); Tight junction protein 3 (ZO-3); G1/S-specific cyclin-D1 (CycD1).

The intestinal barrier is a complex and specialized structure made up of several key components that function synergistically to maintain gut homeostasis and provide protection against harmful substances (Figure 2). The epithelium serves as the crucial boundary separating the gut lumen from the body’s internal milieu and is composed of a single sheet of varied columnar cell types, including enterocytes, goblet cells, and Paneth cells [7]. This diverse cellular composition is crucial for facilitating various gut functions, including nutrient absorption, digestive enzyme secretion, and immune responses [16]. Tight junctions form secure connections between these cells, critically controlling paracellular permeability. This regulation is essential as it permits the selective passage of certain molecules while simultaneously acting as a barrier against pathogens and toxins. These structures are composed of proteins such as occludin, claudins, and zonula occludens protein 1 (ZO-1), which can be modulated by various physiological and pathological stimuli [17]. Dysregulation of tight junctions—often triggered by pro-inflammatory cytokines (e.g., TNF-α, IL-6), gluten peptides, or elevated zonulin—leads to a state of increased intestinal permeability, commonly referred to as “leaky gut.” This breach facilitates the translocation of microbial metabolites, lipopolysaccharides (LPS), and misfolded proteins (e.g., α-synuclein) into the systemic circulation, which in turn drives neuroinflammation and microbiota remodeling, thereby disturbing the gut–brain axis [18,19,20,21,22] (Figure 2). Such mechanisms underscore the intestinal barrier’s pivotal role in both gastrointestinal and neurological health.

Covering the epithelial layer is a thick, stratified mucus layer that acts as the first line of defense against luminal contents. This mucus layer is primarily composed of mucins, which are high-molecular-weight glycoproteins secreted by specialized epithelial cells known as goblet cells (Figure 2). Mucins provide a viscous barrier that traps harmful substances and contributes to the lubrication of the intestinal surface, facilitating the passage of luminal contents [23]. Mucus composition varies regionally: the stomach and colon exhibit dense, continuous layers for mechanical protection, while the small intestine maintains a thinner layer to optimize nutrient absorption [24]. This mucus is enriched with antimicrobial peptides (AMPs, e.g., Paneth cell-derived α-defensins, lysozyme, Reg3 proteins) and secretory IgA, which neutralize pathogens and block epithelial adherence, respectively [25,26]. Beneath this layer lies the gut-associated lymphoid tissue (GALT), a network of immune hubs including Peyer’s patches (PPs) and isolated lymphoid follicles [27]. PPs, concentrated in the ileum, feature B-cell follicles, T-cell zones, and follicle-associated epithelium lined with M cells that sample luminal antigens to initiate adaptive immunity [28,29].

Despite these defenses, the intestinal barrier remains vulnerable to disruption through a few main mechanisms: (1) Epithelial damage: Transient breaches caused by enterocyte apoptosis or injury (e.g., infections, toxins) are rapidly repaired in healthy individuals. However, in individuals with allergies or infections and chronic damage (e.g., in celiac disease or inflammatory bowel disease), the body’s repair mechanisms cannot keep pace with the damage, leading to intestinal contents leaking out and causing a condition known as leaky gut [30]. (2) Certain destructive proteins like gluten directly damage enterocytes or mimic the structure of enterocyte receptors, binding to the cell surface receptor such as CXCR3 and inducing tight junction disassembly [31]. Although some proteins may block normal receptor–carrier interactions, impairing nutrient absorption and barrier integrity [32]. (3) Intestinal cells form tight junctions composed of various proteins that are not permanently solid but can toggle between open and closed states. Disruption of this regulation can result in health issues [33]. One key regulator is zonulin, which is upregulated by gluten exposure in genetically susceptible individuals, triggering tight junction disassembly and paracellular leakage [8,34]. Elevated zonulin levels correlate with conditions like celiac disease and PD, linking gut permeability to systemic inflammation and neurodegeneration. A molecule is critical for opening tight junctions and supporting immune system function.

The intestinal barrier’s integrity relies on synergistic interactions between epithelial cells, tight junctions, mucus, and GALT. Dysregulation at any level via genetic susceptibility, environmental triggers (e.g., gluten) [9], or microbial dysbiosis, which compromises gut homeostasis, fostering systemic inflammation and neuropathology. Targeting these mechanisms offers therapeutic potential for diseases rooted in gut–brain axis dysfunction, such as PD.

## 3. Disruptions in Intestinal Permeability and PD Risk

The integrity of the intestinal epithelial barrier is dependent on tight junction proteins. The dysregulation of these structures, often mediated by pro-inflammatory cytokines (TNF-α, IL-6, IL-1β) and aberrant immune cell activity, leads to a pathological increase in permeability, clinically described as “leaky gut.” [35,36]. Studies suggest that TNF-α suppresses tight junction barrier function via NF-kB (Nuclear factor kappa B) pathway activation, leading to decreased levels of the tight junction protein ZO-1 [37]. The compromised integrity of the tight junction barrier, leading to increased permeability, results from the downregulation of key proteins. Research using Caco-2 cell models has demonstrated that NF-kB-mediated tight junction disruption is associated with elevated expression and activity of myosin light chain kinase (MLCK). The MLCK gene’s promoter harbors a binding site for NF-κB. Through its activation, TNF-α can stimulate the promoter, enhancing MLCK transcription. This process ultimately leads to increased permeability of tight junctions [38,39]. IL-1β, on the other hand, increases tight junction permeability through the activation of the NF-κB pathway, mirroring the effects of TNF-α. Yet the differences are notable. While IL-1β activates MLCK through the extracellular signal-regulated kinases 1/2 (ERK1/2) signaling pathways, it does not affect the ZO-1 protein level as TNF-α does. Instead, IL-1β treatment results in the suppression of occludin, another critical tight junction protein [40].

This increased permeability permits the translocation of luminal antigens, bacteria, and their products, such as LPS, into the systemic circulation. Such translocation can trigger systemic inflammation, fostering the emergence of inflammatory bowel disease (IBD) and metabolic disorders [41,42]. In patients with IBD, altered intestinal permeability is often observed even in asymptomatic individual years before clinical symptoms manifest. For instance, a study reports increased paracellular permeability in patients with quiescent IBD usually exhibit increased paracellular permeability, suggesting that permeability changes can occur independently of overt inflammation [43]. When the mucus layer is compromised due to dysbiosis or inflammation, its protective function diminishes, leading to greater exposure of epithelial cells to harmful substances. This exposure can exacerbate permeability issues initiated by tight junction disruption, allowing pathogens to penetrate more easily and further aggravating inflammation.

Changes in the makeup and density of the intestinal mucus barrier play a critical role in modulating gut permeability. One notable example involves reduced expression of the mucin protein MUC2, which has been closely linked to heightened intestinal leakage. MUC2 is essential for forming a protective mucus gel layer [44], when its synthesis and secretion are decreased, gut microbiota come into direct contact with the epithelial barrier, which results in a greater number of bacteria attaching to the epithelium, highlighting the essential function of MUC2 in preserving the integrity of the mucosal barrier [45].

Additionally, the mucus layer is influenced by the gut microbiota, which produces metabolites that enhance mucin production and support barrier integrity. Pathogenic bacteria, such as enteropathogenic *E. coli* (EPEC), *Citrobacter rodentium*, and *Salmonella typhimurium*, can disrupt the protective mucus barrier, leading to dysbiosis characterized by decreased *Firmicutes* and *Verrucomicrobia*, alongside increased Bacteroidetes and facultative anaerobes, which can adhere to or invade host epithelial cells beneath the mucus layer, further exacerbating inflammation [46]. When the epithelial barrier is compromised, gut-associated lymphoid tissue (GALT) becomes activated, resulting in the release of additional pro-inflammatory cytokines. While this immune response is crucial for combating pathogens, chronic activation can perpetuate inflammation and further disrupt tight junction integrity. The interplay between the immune system and the intestinal barrier is critical, as sustained inflammation can create a cycle where increased permeability triggers immune activation, which in turn causes more damage to the epithelial barrier. The dysregulation of particular immune populations, including T helper 17 (Th17) cells and their cytokines IL-17 and IL-22, plays an important role in disrupting tight junctions and increasing permeability. Th17 cells can dysregulate tight junction protein expression, resulting in compromised barrier integrity. This disruption can create a feedback loop, increased permeability triggers further immune activation, exacerbating intestinal inflammation [47]. On the other hand, IL-17 has been associated with increased gut permeability, as demonstrated by studies where researchers neutralized IL-17 in animal models, leading to heightened intestinal permeability [48].

The passage of harmful substances (e.g., bacterial toxins) from the intestines into the bloodstream, a consequence of increased gut permeability, can initiate a systemic inflammatory response. This mechanism has been associated with the pathogenesis of both PD and IBD. Emerging research underscores the pivotal role of genetic susceptibility factors, such as LRRK2 (leucine-rich repeat kinase 2) variants, which are strongly associated with both PD and IBD [49,50]. LRRK2 mutations may disrupt gut homeostasis, leading to increased permeability and systemic inflammation, which could contribute to the progression of PD via the gut–brain axis [51]. Mutations in the LRRK2 gene, particularly the G2019S and N2081D variants, are the most common genetic contributors to PD and have also been linked to increased incidence of IBD [52]. These shared genetic risks highlight overlapping pathophysiological pathways involving gut–brain axis dysregulation. Higher levels of LRRK2 have been detected in inflamed colonic tissue from Crohn’s disease patients and peripheral immune cells from sporadic PD patients, suggesting that LRRK2 plays a key role in regulating inflammatory processes. Additionally, genome-wide association studies have identified LRRK2 as a common susceptibility locus for both conditions, emphasizing its involvement in immunity, inflammation, and autophagy-related pathways [53]. Recent studies have increasingly altered gut permeability as a mechanistic nexus in diverse conditions, including IBD, metabolic disorders, and neurodegenerative diseases. Heightened gut permeability facilitates the translocation of luminal antigens (e.g., LPS, misfolded α-synuclein) into the systemic circulation, triggering immune activation and autoimmune responses. In PD, such breaches in barrier integrity may exacerbate neuroinflammatory processes by allowing neurotoxic substances to infiltrate the central nervous system (CNS), thereby accelerating the degeneration of dopaminergic neurons.

In addition to the role of LRRK2, the integrity of the intestinal barrier may also be compromised by various other genetic mutations and environmental factors, thereby increasing the risk of PD development. PINK1 and PRKN, two genes involved in mitochondrial quality control and mitophagy, are also associated with early-onset PD symptoms [54]. Loss-of-function mutations in these genes impair mitochondrial homeostasis in intestinal epithelial cells, rendering them more susceptible to oxidative stress and apoptotic signaling, which can weaken the epithelial barrier [55,56]. Recent study using PINK1-deficient mouse models have provided compelling evidence that mitochondrial dysfunction can initiate PD-related pathology through the gut. Loss of PINK1 impairs the clearance of damaged mitochondria, leading to the release of mitochondrial DAMPs that activate innate immune pathways, including NF-κB and the NLRP3 inflammasome [57]. In a PD mouse model triggered by *Citrobacter rodentium* infection, PINK1 knockout mice exhibited exaggerated innate immune activation and upregulation of pro-inflammatory cytokines (e.g., TNF-α, IL-1β). This dysregulated environment promoted robust activation of T cells, which in turn amplified epithelial stress through cytokine secretion, notably IFN-γ and IL-17A. These cytokines are known to disrupt tight junction proteins and weaken epithelial integrity, leading to increased intestinal permeability [58]. These findings suggest that mitochondrial dysfunction may directly or indirectly compromise intestinal epithelial integrity, thereby creating a permissive environment for systemic inflammation and neurodegeneration via the gut–brain axis. Elucidating the interplay between LRRK2-mediated pathways, mitochondrial dysfunction, gut permeability, and neuroinflammation is critical for identifying novel therapeutic targets to disrupt disease progression in both PD and IBD.

## 4. The Interplay Between Blood–Brain Barrier Integrity and Neurodegenerative Diseases

Intestinal permeability allows harmful substances like bacteria, toxins, or inflammatory molecules to enter the bloodstream, triggering systemic inflammation that can exacerbate neuroinflammation. This inflammation may compromise the BBB, which normally protects the brain from harmful agents. Converging experimental and clinical data support a link between increased intestinal permeability and compromised BBB integrity, largely mediated by gut microbiota dysbiosis and its downstream effects. For example, increased BBB permeability has been observed in mouse models of colitis induced by DSS or TNBS, as well as in models of antibiotic-induced dysbiosis [59,60,61,62]. Germ-free mice display heightened BBB permeability accompanied by reduced tight junction proteins (occludin, claudin-5), a phenotype reversed by microbiota colonization [63]. In vitro, gut-derived LPS induces endothelial injury via microglial activation [64]. In humans, elevated plasma markers of gut (zonulin, LPS) and BBB (claudin-5) permeability correlate with psychiatric disorders [65], suggesting that gut barrier dysfunction facilitates translocation of microbial products that drive neuroinflammation and BBB breakdown [66]. The breakdown of the BBB has profound pathological consequences that significantly contribute to neurodegenerative diseases such as PD and Alzheimer’s disease (AD) [67,68]. When BBB integrity is compromised, neurotoxic molecules, immune cells, and inflammatory mediators infiltrate the CNS, disrupting neurotransmitter balance and impairing synaptic function. Pro-inflammatory cytokines such as TNF-α and IL-1β have been shown to weaken BBB tight junctions, leading to increased permeability [69]. Furthermore, the BBB restricts the transport of neurotrophic factors (e.g., Brain-Derived Neurotrophic Factor, Neurotrophin-3, and Nerve Growth Factor), which are essential for neuronal survival and plasticity. The influx of harmful substances into the brain can lead to direct neuronal injury as well. Research has shown that exposure to blood-derived factors promotes neuroinflammation and tissue injury that can lead to neuronal death [70], contributing to the progressive loss of neuronal populations characteristic of neurodegenerative diseases [71]. Ultimately, these processes lead to neuronal death. This is exacerbated by chronic inflammation resulting from BBB dysfunction, which initiates a cascade of mitochondrial damage, oxidative stress, and neurovascular disruption that accelerates neurodegeneration [72]. However, the precise pathways vary by disease stage and remain under active investigation.

Interestingly, the intestinal epithelial barrier (IEB) and BBB share significant structural and functional similarities, particularly in their reliance on tight junction proteins such as ZO-1, claudin-5, and occludin [73]. Both barriers regulate selective permeability, protecting their respective environments while permitting the transport of essential nutrients and molecules. Emerging evidence suggests that inflammatory mediators known to disrupt the intestinal barrier, including TNF-α, IL-1β, zonulin, IFN-γ, and IL-17A, may also compromise BBB integrity through analogous mechanisms, although direct evidence in PD is still developing [38,69,73]. This establishes a plausible connection between leaky gut syndrome and PD, whereby compromised BBB function could exacerbate neuroinflammatory and neurodegenerative processes. A study by Rahman et al. [73] further supports this connection, demonstrating that exposure to zonulin, IFN-γ, or IL-17A rapidly alters the localization of tight junction proteins, including ZO-1 and Claudin-5, in both barriers. This process involves a rapid disassembly of tight junctions and significant depolymerization of the peri-junctional F-actin cytoskeleton [73]. Further, both barriers show increased permeability to a fluorescein isothiocyanate (FITC)-dextran tracer within 1 h of exposure to these mediators, highlighting the rapid and significant impact on barrier function [73]. Given these shared mechanisms, alterations in IEB permeability due to inflammatory processes may similarly affect BBB integrity. This establishes a logical connection between leaky gut syndrome and PD, as compromised BBB function can exacerbate neuroinflammatory and neurodegenerative processes. Obviously, in PD, particularly in individuals with LRRK2 mutations, this dual barrier dysfunction amplifies the transport of pro-inflammatory signals and potentially misfolded proteins, such as alpha-synuclein, from the gut to the brain [3], thereby promoting neuronal damage and disease progression. Thus, it is hypothesized that dual barrier dysfunction may create a vicious cycle, potentially amplifying the transfer of inflammatory signals and misfolded α-synuclein from the periphery to the brain.

## 5. Leaky Gut and Gut Microbiota Dysbiosis

In Parkinson’s disease (PD), a destructive feedback loop can emerge where increased permeability of both the gut barrier and the blood–brain barrier (BBB) mutually exacerbate neuroinflammation and accelerate neuronal loss. Gut dysbiosis, an alteration of gut microbial homeostasis, can disrupt the BBB, allowing harmful substances and inflammatory molecules to enter the brain and trigger neuroinflammation and neuronal damage [63,74,75]. Increased intestinal permeability and gut microbiota dysbiosis are closely interconnected in a bidirectional relationship that contributes to numerous inflammatory and metabolic diseases. Dysbiosis is a state of microbial imbalance defined by a decline in beneficial bacteria and a concomitant proliferation of potentially harmful pathobionts. Under these conditions, epithelial barrier function is weakened through diminished short-chain fatty acids (SCFAs), impaired tight-junction protein expression, and promotion of pro-inflammatory mediators such as LPS [16,42,74,76,77]. This leads to increased permeability of the gut lining, allowing microbial products to translocate into systemic circulation and trigger immune activation. Conversely, a compromised intestinal barrier further exacerbates dysbiosis by altering the gut environment, promoting inflammation, and impairing host-microbe signaling. Increased intestinal permeability, also known as “leaky gut,” involves the weakening of the intestinal lining. This breakdown enables the passage of harmful substances—including bacterial toxins, microbial fragments, and undigested dietary compounds—into the systemic circulation. This condition is fundamentally connected to an imbalance in the gut’s microbial community. This state of dysbiosis disrupts the ecosystem’s normal function, resulting in a dysregulated immune response and subsequent inflammatory processes [42]. Dysbiosis degrades protective mucus layers, weakens tight junctions between epithelial cells, and triggers the release of pro-inflammatory cytokines (e.g., TNF-α, IL-6), thereby exacerbating intestinal permeability [16]. Consequently, these interconnected processes can initiate systemic inflammation and are implicated in the pathogenesis of autoimmune disorders (e.g., IBD), metabolic diseases (e.g., type 2 diabetes), and neurological conditions. While research continues to elucidate causal relationships, therapeutic strategies targeting microbiota restoration (e.g., probiotics, dietary fiber) show promise in mitigating both dysbiosis and barrier dysfunction. The vicious cycle of barrier disruption and microbial imbalance underlies conditions such as IBD, obesity, and neurodegenerative disorders, and highlights the gut barrier as a key therapeutic target [7].

Research consistently demonstrates that heightened intestinal permeability correlates with reduced populations of beneficial gut bacteria, underscoring the microbiome’s critical role in gut health. Dysbiosis directly impacts immunity, as gut microbiota regulates about 10% of genes linked to immune and metabolic functions in intestinal epithelial cells [78]. For example, Cani et al. (2008) observed that mice on high-fat diets exhibited increased permeability alosngside diminished *Lactobacillus* and *Bifidobacterium*, enabling LPS translocation into the bloodstream, triggering systemic inflammation and insulin resistance [79]. This synergy between dysbiosis and barrier dysfunction exacerbates metabolic disorders. Further elucidating this interplay, Liu et al. (2023) revealed that *Akkermansia muciniphila* depletion in mice elevated permeability and inflammation, while its restoration via diet improved barrier integrity [80]. Notably, strain-specific effects of *Akkermansia* were tied to functional genes governing surface protein synthesis [80]. Human studies are not fully consistent, but meta-analyses generally report increased *Akkermansia* in PD rather than decreases. These discrepancies likely stem from variables like disease stage, geography, diet, medications (e.g., levodopa), or methodological differences. Complementing these insights, *Bifidobacterium* strains (e.g., *B. bifidum*) enhance tight junction proteins via PPAR-γ pathway activation [81], reinforcing that microbial diversity is essential for barrier resilience. On the contrary, in the context of gut microbiota dysbiosis, probiotics have shown considerable promise in enhancing intestinal barrier function. Specific probiotic strains, such as *Bifidobacterium breve CNCM I-5644*, have demonstrated the ability to prevent intestinal hyperpermeability by upregulating tight junction proteins and reducing pro-inflammatory markers in both in vitro studies and animal models of IBS [82]. For example, research has indicated that this strain can enhance the expression of cingulin and tight junction protein 1 in gut barrier-compromised mouse models to maintain tight junction integrity [82]. However, this study does not suggest that *CNCM 1-5644* would exhibit similar efficacy in the human intestinal environment.

Further research, including clinical trials, is required to investigate the probiotic’s ability to establish and maintain a stable colony in the human gut and its therapeutic potential and safety in human subjects. On the clinical side of the research, a randomized controlled trial investigated the effects of a multi-strain probiotic mixture, including *Lactobacillus paracasei HII01*, *Bifidobacterium breve*, and *Bifidobacterium longum*, on various health parameters in elderly participants. The study found that supplementation with these probiotics significantly improved intestinal barrier function, enhancing permeability by up to 48%. Additionally, the intervention led to a notable increase in high-density lipoprotein (HDL) cholesterol levels and improved obesity-related anthropometric measurements and SCFA levels. Although this study does not specifically investigate PD, its findings on increased intestinal permeability suggest that probiotic supplementation could potentially contribute to improvements in PD by enhancing gut barrier function and dampening systemic inflammation [83].

## 6. PD and Gut Microbiota Dysbiosis

Increased intestinal permeability initiates a series of events that contribute to neuroinflammation. Disruption of the gut barrier leads to microbiota dysbiosis, triggering chronic inflammation and the release of pro-inflammatory cytokines. These inflammatory signals and microbial byproducts enter circulation, affecting the CNS and contributing to the neuroinflammation processes. PD is increasingly linked to gut microbiota dysbiosis, characterized by altered microbial composition, such as reduced *Prevotella* and *Faecalibacterium* and increased *Enterobacteriaceae*, which may contribute to disease pathology via the gut–brain axis. Dysbiosis can enhance gut permeability, leading to systemic inflammation and potentially promoting misfolded alpha-synuclein propagation from the gut to the brain through the vagus nerve, exacerbating neuroinflammation and motor symptoms [79]. Gastrointestinal symptoms like constipation often precede PD diagnosis, supporting early gut involvement [84]. Microbial metabolites, such as SCFAs, may modulate dopamine metabolism and immune responses, influencing PD progression [85].

Altered intestinal permeability sets the stage for gut microbiota dysbiosis, where the natural balance of beneficial and harmful bacteria is disrupted. This dysbiosis can initiate a cycle of inflammation that not only affects the gut but also has systemic implications. As the gut’s barrier function deteriorates, inflammatory signals and microbial byproducts can enter the bloodstream, influencing the CNS (Figure 2). Over time, this continuous state of dysbiosis and inflammation may contribute to the neurodegenerative processes seen in PD. Tight junction components—such as occludin, claudins, and junctional adhesion molecules (JAMs)—play essential roles in maintaining barrier function. Alterations in the expression of claudin-1, claudin-3, and claudin-5, all key sealing proteins, or the upregulation of pore-forming claudin-2, can significantly alter paracellular permeability [86]. Notably, a critical player in this process is the tight junction protein ZO-1, which regulates both intestinal and BBB integrity. Dysfunction of ZO-1 disrupts the tight junctions between epithelial cells in the gut and endothelial cells in the brain’s vasculature, effectively weakening these barriers (Figure 2). This loss of structural integrity may enable the uncontrolled passage of harmful substances, leading to neuroinflammation and oxidative stress, alongside α-synuclein misfolding and aggregation—key features of PD pathology. The resulting leakage of inflammatory signals and microbial components into the brain underscores a vital link between leaky gut and PD pathogenesis (Figure 2). The sustained inflammatory response can accelerate dopaminergic neuron degeneration in the substantia nigra, exacerbating motor and cognitive symptoms characteristic of PD. Additionally, microbial metabolites and systemic inflammation may impair mitochondrial function and protein homeostasis, further amplifying neuronal vulnerability. Understanding these links is crucial for unraveling how disturbances in gut health may drive or exacerbate Parkinson’s pathology.

Dysbiosis caused by gut barrier dysfunction critically influences the CNS through the gut–brain axis, altering neurotransmitter metabolism and amplifying neuroinflammatory pathways. Clinical and translational studies consistently report that patients with PD exhibit significant changes in gut microbiota composition compared to healthy controls, characterized by depletion of anti-inflammatory taxa and expansion of pro-inflammatory species. An early human study conducted by Petrov et al. (2016) reported that PD patients had a marked reduction in beneficial bacteria like *Faecalibacterium prausnitzii* and members of the *Lachnospiraceae* family, which are known key butyrate producers with anti-inflammatory properties [87]. In contrast, a meta-analysis of ten 16S microbiome datasets highlights the unexpected enrichment of *Akkermansia* and *Bifidobacterium*, typically beneficial probiotics and raises questions about their role in PD pathology. While *Akkermansia* is known to support gut barrier integrity and *Bifidobacterium* have anti-inflammatory properties, their presence in a pro-inflammatory PD microbiome suggests a potential compensatory response or an altered function in disease conditions. These findings underscore the complexity of gut microbial dynamics in PD and the need for further investigation into their mechanistic role [88]. Gastrointestinal symptoms, such as constipation and dysbiosis, frequently manifest before cognitive impairment in PD, suggesting that gut health could be an early marker of disease progression. A clinical analysis by Jones et al. explored the relationship between the frequency of GI symptoms and PD progression. Their findings revealed that nearly 70% of PD patients reported GI issues. Moreover, a significant correlation (*p* < 0.01) was identified between the severity of GI symptoms and motor symptoms in PD patients, indicating that as GI disturbances intensify, motor symptoms also worsen [89]. This highlights a potential link between gut health and motor function in PD.

The mouse model study by Sampson et al. [90] demonstrates that gut microbiota plays a critical role in regulating motor deficits and neuroinflammation in a mouse model of PD. Using Thy1-α-Syn (ASO) mice that overexpress α-Syn, the study demonstrated that gut microbiota is essential for the manifestation of standard motor deficits, including impaired performance on tasks like beam walking, pole climbing, sticky tape removal, and reflexive hindlimb clasping. Germ-free ASO mice exhibited significantly reduced motor deficits compared to their conventionally raised counterpart. Interestingly, Germ-free ASO mice also displayed less α-Syn aggregation in brain regions like the caudoputamen and substantia nigra, which are affected in both the mouse model and human PD. The study also showed that giving germ-free mice certain microbial metabolites by mouth triggered neuroinflammation and motor deficits, indicating that signals originating in the gut can directly impact brain function. Moreover, when ASO mice were colonized with microbiota derived from PD patients, motor impairment was exacerbated relative to mice receiving healthy-donor communities, indicating that PD-linked dysbiosis can influence disease progression. These findings provide compelling evidence that alterations in gut microbiota may profile as potential modifiers of PD course and highlight gut–brain crosstalk in the pathogenesis of neurodegenerative disorders [90].

## 7. Potential Therapeutic Approaches

Parkinson’s disease treatment encompasses a range of approaches aimed at managing symptoms and potentially slowing progression, including pharmacological therapies like levodopa/carbidopa, dopamine agonists, and MAO-B inhibitors for motor symptom relief; surgical options such as deep brain stimulation (DBS) for advanced cases; non-pharmacological interventions like physical, speech, and occupational therapies to enhance mobility and quality of life; and emerging therapies such as gene therapy, stem cell therapy, probiotics (microbiota), immunotherapy targeting alpha-synuclein, and focused ultrasound, which are under investigation for neuroprotection or disease modification, alongside lifestyle interventions like diet and cognitive behavioral therapy for holistic management [91]. The following discusses the more detailed potential approaches.

### 7.1. Short-Chain Fatty Acids

SCFAs are primary metabolites produced by the gut microbiota and serve as crucial signaling molecules that facilitate communication between the gut and the central nervous system, specifically the brain. Clinical studies have commonly reported gut microbiota dysbiosis and SCFA deficiency in PD populations [92,93,94]. They may significantly influence PD by modulating inflammatory responses, ensuring neuronal survival, and maintaining the integrity of the gut barrier and BBB [66,95]. The reduced level of SCFAs serves as a key example of the complex interplay between gut permeability, gut microbiota dysbiosis, and progression of PD. These metabolites, including acetate, propionate, and butyrate, are essential for maintaining intestinal barrier integrity and overall metabolic homeostasis. SCFAs activate specific G-protein coupled receptors (GPRs) located on the cell surface, triggering a series of intracellular signaling events. These pathways involve the activation of phospholipase C, mitogen-activated protein kinases (MAPKs), phospholipase A2, and the transcription factor NF-κB. Key receptors mediated by SCFAs are GPR41 (FFAR3), GPR43 (FFAR2), and GPR109A [96]. GPR43 has a high affinity for acetate and propionate [97,98], while GPR41 prefers butyrate. These receptors are widely expressed in brain endothelial cells, neurons, and immune cells, making them essential for SCFAs in regulating CNS function. After SCFAs are produced by gut microbiota, the vast majority are taken up by colon epithelial cells [99], and only a limited fraction entering systemic circulation. From the bloodstream, SCFAs can potentially influence the brain, which is supported by the presence of proton-coupled monocarboxylate transporters (MCTs) and sodium-coupled monocarboxylate transporters (SMCTs) in brain endothelial cells, astrocytes, and neurons [95]. In addition, the uptake of SCFAs into the brain via MCTs has been previously demonstrated [95,100].

SCFAs treatment has been reported to nourish and strengthen the intestinal epithelial barrier by promoting tight junction protein assembly [76,101,102,103]. It has also been reported that monocolonization with the butyrate-producing *Clostridium tyrobutyricum*, the acetate-/propionate-producing *Bacteroides thetaiotaomicron*, or oral gavage of sodium pyruvate helps germ-free mice restore BBB integrity by increasing the expression of the tight junction protein occludin in the frontal cortex and hippocampus [63]. Recent evidence suggests that SCFAs may help treat PD by improving gut health and mitigating symptoms. In a 2025 pilot study, 10 patients with PD followed a high-fiber diet for 4 weeks, which increased SCFA levels (e.g., butyrate), improved gastrointestinal symptoms, and modestly reduced motor impairment, although overall microbiome composition was not fully restored [104]. In vivo mouse studies have shown that SCFAs, particularly butyrate and propionate, possess neuroprotective properties. For instance, studies in animal models have shown that butyric acid can enhance motor performance [105] and reduce degeneration of dopaminergic neurons [106]. In contrast, propionic acid demonstrates protective effects on neurons under stress [107], suggesting that SCFAs may help mitigate PD-related neurodegenerative processes [108]. The deficiency of SCFAs can lead to several detrimental effects that may exacerbate PD. First, SCFAs are known for their anti-inflammatory properties; they can directly block inflammatory signaling pathways, such as NF-kB and MAPK, within the CNS while contributing to the reduction in neuroinflammation by decreasing peripheral inflammation and reinforcing the BBB [109,110]. A reduction in SCFAs can compromise this protective effect, potentially leading to increased brain inflammation and neuronal damage. Additionally, SCFAs are crucial for maintaining the integrity of the gut barrier. A decrease in SCFAs can result in a weakened intestinal barrier, allowing toxins and inflammatory cytokines to translocate into the systemic circulation and traffic to the brain, further aggravating neuroinflammation and neuronal dysfunction [92]. This disruption can create a vicious cycle, as increased inflammation can lead to further dysbiosis and a continued decline in SCFA production. Clinical metabolomic studies support the observation of reduced SCFA production in PD. Seminal work by Unger et al. first reported significantly lower fecal SCFA concentrations in PD patients compared to healthy controls [93]. This finding has been expanded upon in more recent analyses, which also report a correlation between diminished SCFA levels and earlier disease onset, alongside elevated fecal calprotectin levels suggesting concomitant gut inflammation [92].

Furthermore, SCFA levels were positively correlated with age at PD onset, suggesting that diminished SCFA production may contribute to the earlier manifestation of the disease. The concurrent increase in fecal calprotectin further supports the presence of heightened intestinal inflammation in PD, particularly in a sex-dependent manner, as baseline levels are higher in males than in females. While plasma and stool inflammatory markers were not strongly correlated with SCFA levels, the microbiota’s composition and diversity were closely tied to both inflammation and gut permeability markers, such as zonulin and Claudin-5 [111]. These findings highlight a disrupted microbiota-host interaction in PD, where reduced SCFA production and increased gut inflammation may contribute to exacerbating disease progression and symptom severity. As the gut microbiota becomes increasingly imbalanced, the diversity and abundance of SCFA-producing bacteria diminish, resulting in even lower levels of these protective metabolites. This decline exacerbates intestinal permeability, allowing more toxins and inflammatory mediators to enter the bloodstream, which in turn heightens neuroinflammation and contributes to neuronal damage.

The clinical evidence for SCFAs directly treating PD remains limited, with most research at the preclinical stage. To date, clinical evidence directly assessing the therapeutic potential of SCFAs in PD remains limited. Only one identified clinical trial (NCT05446168) directly investigates SCFA supplementation, utilizing oral tributyrin, a butyrate prodrug, to evaluate gut and brain uptake via [^11^C] butyrate PET imaging in PD patients [112]. Beyond this, several indirect approaches aim to enhance endogenous SCFA production through dietary or microbiome interventions. Notably, a study (NCT04512599) investigated prebiotic supplementation in PD patients, demonstrating improvements in intestinal permeability and increases in SCFA levels, alongside reductions in fecal markers of gut inflammation such as zonulin and calprotectin [113]. Similarly, (NCT02784145) explored nutritional strategies designed to modulate the microbial populations within the GI tract of individuals with Parkinson’s disease, with reported outcomes including altered microbial profiles, increased abundance of SCFA-producing taxa, and improved gastrointestinal function [114]. Collectively, these findings suggest that while direct SCFA trials are in their infancy, indirect strategies to modulate SCFA levels through diet and microbiota manipulation are more established and demonstrate potential for therapeutic benefit.

### 7.2. Fecal Microbiota Transplantation

Fecal microbiota transplantation (FMT) is increasingly recognized as a potential therapy for rebalancing the intestinal microbiota and improving intestinal barrier integrity. Boehme et al. [115] demonstrated that transplanting feces of young mice into aged recipients through fecal bacterial transplantation and found that a series of related cognitive and behavioral disorders in aging mice were improved, confirming the possible link between intestinal flora and neurological diseases. Similarly, in alcohol-exposed C57BL/6J mice, a study found that FMT effectively restored gut barrier function by reducing intestinal permeability markers and enhancing the expression of tight junction proteins that maintain the integrity of the intestinal epithelium [116]. Although the current literature does not specifically focus on the impact of FMT on gut permeability, it consistently highlights the positive effects of FMT on the regulation of the patient’s gut microbiota [117,118,119,120]. While more research is needed to fully understand its mechanisms and long-term effects, several studies suggest that FMT may be beneficial for conditions associated with increased gut permeability through gut microbiota regulation. This approach is based on the premise that a balanced gut microbial community is essential for maintaining intestinal barrier function. In cases of dysbiosis, where the gut microbiome is disrupted, FMT may help correct the imbalance and improve gut barrier integrity. FMT is emerging as a transformative clinical strategy in PD, bridging mechanistic insights into the gut–brain axis with actionable therapeutic applications. Recent clinical trials underscore its potential; for instance, a pilot study by Cheng et al. (2023) demonstrated that PD patients receiving FMT from healthy donors exhibited not only improved gastrointestinal function (e.g., reduced constipation) but also significant alleviation of motor symptoms [121]. These findings align with the hypothesis that restoring a balanced gut microbiome via FMT mitigates gut-derived neuroinflammation and α-synuclein aggregation. Mechanistically, FMT replenishes beneficial taxa (e.g., *Blautia, Roseburia*) that produce neuroprotective SCFAs while suppressing pro-inflammatory microbes linked to intestinal permeability and systemic inflammation.

Animal studies have demonstrated that FMT can reverse PD-like symptoms and neuroinflammation in models induced by toxins and α-synuclein overexpression. Initial clinical case reports and pilot trials have reported improvements in both motor and non-motor symptoms, including constipation, mood disturbances, and rigidity, following FMT in PD patients. For instance, a study involving 56 subjects with mild-to-moderate Parkinson’s found that FMT treatment yielded two primary outcomes: a significant reduction in autonomic symptoms related to PD and a measurable recovery of healthy gut bacteria, outcomes not observed in the placebo cohort. However, the study did not observe notable enhancements in motor symptoms, suggesting a selective benefit of FMT on autonomic functions. These findings underscore the complexity of FMT’s role in PD management, indicating that while it may alleviate certain non-motor symptoms, its impact on motor function remains uncertain [121]. Despite the need for further research, FMT holds promise as a potential therapy for conditions characterized by increased intestinal permeability. By targeting the underlying dysbiosis and restoring a healthy gut microbiome, FMT may offer a novel strategy for improving gut barrier function and overall GI and neurological health. Despite these encouraging results, standardized protocols for FMT administration, donor screening, and long-term safety monitoring are lacking. Larger, randomized controlled trials are needed to validate these findings and clarify the mechanistic links between microbial restoration and neuroprotection in PD. Nonetheless, FMT represents a promising, gut-directed intervention with disease-modifying potential in PD.

### 7.3. Induced Pluripotent Stem Cell (iPSC)-Based Therapy: A Personalized Regenerative Strategy

Induced pluripotent stem cell (iPSC)-based therapy has emerged as a transformative approach in the treatment and modeling of PD, with profound implications for understanding and addressing key pathological drivers, including dopaminergic neurodegeneration, neuroinflammation, α-synuclein aggregation, and gut–brain axis dysfunction [15]. First introduced by Takahashi and Yamanaka in 2006, iPSCs are generated by reprogramming somatic cells—such as fibroblasts or blood cells—into a pluripotent state [122]. Unlike conventional dopaminergic therapies that only mitigate motor symptoms, iPSC-derived neuronal replacement strategies aim to restore lost neural function, while also providing personalized platforms to probe the mechanisms underlying disease progression. From a therapeutic standpoint, human iPSC-derived dopaminergic progenitors have shown encouraging preclinical success in restoring motor function in PD models.

Recent breakthroughs in iPSC-based therapy for PD include the successful transplantation of iPSC-derived dopaminergic neurons into patients, demonstrating improved motor function and graft survival in early-phase clinical trials [123]. Researchers have optimized differentiation protocols to generate midbrain-specific dopaminergic progenitors with enhanced purity and functionality, reducing tumorigenic risks [124]. In a recent study, iPSCs generated from PD patients were differentiated into midbrain dopaminergic cells (mDACs) using a clinically compliant protocol and implanted into animal models. These cells demonstrated long-term survival, absence of tumorigenicity, and the ability to integrate into host circuits. Importantly, variability in functional recovery was found to correlate with dopaminergic fiber density, emphasizing the need for precise graft characterization before clinical use [125]. Autologous iPSC therapies now employ HLA-haplobanked cell lines (e.g., CiRA’s initiative) to enable “off-the-shelf” allogeneic transplants with minimal immune rejection. CRISPR-edited iPSCs have corrected PD-associated mutations (e.g., LRRK2 G2019S) while integrating neuroprotective genes like GDNF for combined cell replacement and neurotrophic support. Advanced 3D midbrain organoid models derived from patient iPSCs are accelerating drug discovery and mechanistic studies of α-synuclein pathology, with recent success in identifying autophagy-enhancing compounds [126]. These advancements are complemented by improved delivery systems using biomaterial scaffolds to enhance neuronal integration and survival post-transplantation.

Building on this, comparative studies have explored the differentiation efficiency and safety profiles of dopaminergic neurons derived from human embryonic stem cells (hESCs), human iPSCs, and non-human primate iPSCs. Optimized protocols that enrich ventral midbrain identity and remove undifferentiated cells have improved the safety and functional consistency of candidate cell therapies [127]. Another preclinical evaluation using clinical-grade iPSC-derived dopaminergic progenitors confirmed their ability to survive, express midbrain markers, and improve motor symptoms without forming tumors, reinforcing their translational potential [128].

Equally important, this review believes that iPSC-based systems can serve as a unique window into patient-specific disease mechanisms. These models preserve genetic, epigenetic, and environmental susceptibility features, which may allow researchers to study how gut-derived inflammatory signals, microbial metabolites, or α-synuclein strains influence neuronal function across individuals. For example, neurons derived from different PD patients may show differential vulnerability to LPS, zonulin, or SCFA depletion, factors implicated in gut permeability and systemic inflammation. This makes iPSCs an ideal tool to dissect how gut–brain axis dysfunction manifests at the cellular level in PD.

The dual utility of iPSCs, as both a regenerative therapy and a high-resolution model system, supports their integration into the broader framework of personalized medicine. More ambitiously, iPSC-derived dopaminergic progenitors have been shown in preclinical models to survive, integrate into host circuitry, and restore motor function following transplantation. As emphasized in recent reviews, the convergence of iPSC technology with gut–brain axis research opens new avenues for stratifying PD patients, identifying therapeutic windows, and evaluating interventions tailored to individual pathophysiological profiles [129]. In the context of gut-permeability–driven PD, iPSC-derived neurons can be exposed to cytokines (e.g., TNF-α, IL-1β), microbial ligands (e.g., LPS), or metabolite shifts (e.g., SCFA depletion) in vitro to model neuroinflammatory stressors stemming from intestinal barrier dysfunction. Such systems can help test the downstream neuronal effects of microbiota-targeted therapies, zonulin antagonists, or diet-based interventions in a humanized, controlled setting. This makes iPSCs not merely a treatment modality, but a platform for understanding how peripheral inflammation and gut pathology translate into central dopaminergic neurodegeneration.

### 7.4. Microbiome Modulation: From Conventional Therapies to Precision Medicine

Recent research has focused on the gut microbiome and its metabolites as novel therapeutic targets, exploring their role in modulating neuroinflammation, gut barrier integrity, and dopaminergic signaling. Humans and their commensal microorganisms have co-evolved in a reciprocal partnership. Accordingly, host–microbe symbiosis is essential for maintaining health and influencing the likelihood of disease development [130]. Many studies have evaluated the therapeutic potential of gut microbial interventions across multiple diseases [131]. In most cases, existing evidence remains inconclusive as to whether microbial shifts initiate disease or result from it, and whether therapeutic modification of the gut microbiome can effectively manage or cure the disorder [132]. Various lifestyle changes through alternative treatments—including FMT, dietary modifications, and the use of probiotics, prebiotics, and symbiotics—show promise for counteracting gut microbial dysbiosis. These approaches may help promote healthy microbial populations and have minimal long-term negative effects [133,134,135,136].

The landscape of therapeutic approaches for managing gut health and its implications for PD is evolving rapidly. While these conventional therapies have provided some benefits while addressing gut dysbiosis and associated symptoms, they often lack the precision to effectively target the complex interactions within the gut microbiome and the gut–brain axis. However, recent innovations in microbiome research are paving the way for more targeted and effective interventions that can be tailored to individual patient needs. One of the most promising strategies is the use of in situ targeted mutagenesis of gut bacteria, which allows for precise genetic modifications to enhance gut health and potentially slow the progression of neurodegenerative diseases like PD. Clinical evidence for microbiome modulation in PD is emerging, with studies highlighting the gut–brain axis as a key therapeutic target. Observational data show that PD patients often exhibit gut dysbiosis, characterized by reduced levels of beneficial bacteria such as *Prevotella* and *Faecalibacterium prausnitzii* and increased pro-inflammatory species, including *Enterobacteriaceae* [87]. A 2023 randomized controlled trial demonstrated that FMT improved motor symptoms and constipation in PD patients by restoring microbial balance, with effects lasting up to 6 months [121]. Probiotic interventions, such as *Lactobacillus* and *Bifidobacterium* strains, have shown promise in small trials by reducing neuroinflammation and improving non-motor symptoms like cognitive impairment and gastrointestinal dysfunction [137]. Preclinical studies also suggest that SCFAs produced by gut bacteria, such as butyrate, may protect dopaminergic neurons [112,113]. However, small sample sizes and variability in microbial responses limit definitive conclusions, and larger clinical trials are needed to validate these findings and develop precision microbiome therapies for PD.

The innovative research conducted by Brödel et al. [138] presents a transformative method for modifying gut bacteria directly within their natural environment, addressing significant limitations of traditional microbiome manipulation techniques. The study introduces a phage-derived particle capable of delivering genetic modifications to specific bacterial populations in the gut, a breakthrough that could reshape microbiome-targeted therapies [138]. By engineering a phage-derived particle to deliver base editors to Escherichia coli colonizing the mouse gut, the researchers established a novel and precise tool for investigating bacterial gene functions [138]. Remarkably, the genetic modifications were stable for at least 42 days post-treatment, demonstrating the technique’s potential for durable therapeutic applications. This method bridges basic research and clinical innovation, offering a precise, controlled approach to regulating gut permeability and addressing conditions linked to gut dysbiosis.

The therapeutic implications of this method are vast, particularly for conditions where gut dysbiosis plays a central role. For example, in neurodegenerative diseases like PD, where increased gut permeability and systemic inflammation are linked to disease progression, this technique could be employed to target and modify bacterial genes contributing to neurotoxic metabolite production or inflammatory mediator release. Such interventions can restore gut barrier integrity, mitigate neuroinflammation, and support neurological health.

### 7.5. Genetic Stratification: Towards Precision Medicine in PD

The clinical presentation and disease progression vary considerably among individuals, reflecting diverse underlying pathophysiological mechanisms. Traditional treatment strategies, particularly dopamine replacement therapy, provide symptomatic relief but fail to address this heterogeneity or modify disease progression. As a result, there is a growing need for personalized therapeutic approaches that tailor interventions to each patient’s unique genetic, molecular, and clinical profile. Levodopa remains the gold standard for symptomatic treatment of PD, particularly for improving motor function. However, long-term levodopa therapy is associated with complications such as motor fluctuations, dyskinesias, and reduced therapeutic duration of benefit. These motor complications typically emerge in over 50% of patients after five years of continuous treatment [139]. While earlier research identified these challenges, more recent reviews emphasize the need for comprehensive treatment strategies that extend beyond dopaminergic replacement [140].

Genetic susceptibility factors, such as LRRK2 variants, PINK1, and PRKN are strongly associated with both PD and IBD, highlighting shared pathogenic pathways involving gut permeability, immune activation, and neuroinflammation are strongly associated with both PD and inflammatory conditions like IBD, highlighting shared pathways involving gut permeability and neuroinflammation. Beyond the previously mentioned genetic factors, multiple genes have been implicated in familial PD, including SNCA and DJ-1, which play key roles in α-synuclein aggregation, oxidative stress response, and cellular homeostasis [141]. In addition, genome-wide association studies (GWAS) have identified over 90 genetic loci that modestly increase the risk of PD in sporadic cases [142]. Notable among these are GBA, involved in lysosomal function [143]; MAPT, linked to microtubule dynamics [144]; and TMEM175 and BST1, which influence autophagy and immune regulation [145]. Although no single gene accounts for all cases, the expanding list of risk and modifier genes reflects the complex polygenic architecture of PD, combining rare, high-impact mutations with common, low-penetrance variants. Therefore, stratifying patients based on their genetic makeup can inform targeted interventions aimed at modifying disease risk and progression. Such stratification could guide decisions on microbiota-targeted therapies, immune-modulating treatments, or even the suitability of iPSC-derived therapies for particular patient subgroups. Ultimately, integrating genetic stratification with microbiome and regenerative approaches represents a pathway toward more precise, individualized treatment strategies for PD.

### 7.6. Current and Emerging Therapies Targeting Gut Permeability

While traditional PD treatments primarily focus on managing motor symptoms, emerging research highlights the potential of therapies targeting the gut–brain axis. These approaches aim to address intestinal permeability and its associated impact on PD pathology. The following interventions represent a spectrum of current and potential treatments that may influence gut health and, by extension, neurological outcomes in PD patients (Table 1).

Gut permeability is a critical factor in the development of inflammatory, metabolic, and neurodegenerative diseases, including PD, due to its role in enabling systemic inflammation via microbial translocation. Current therapies—such as probiotics, prebiotics, SCFAs, and zonulin antagonists like larazotide acetate—aim to restore intestinal barrier integrity by enhancing tight junction protein expression and reducing gut inflammation [17,18,19]. Emerging strategies, including FMT, glutamine supplementation, and phytochemicals like curcumin, offer broader anti-inflammatory and mucosal-healing benefits but may lack patient-specific precision [7,8,9,10,12,13,16]. A major breakthrough in this field is the development of genetically engineered microbiota; for instance, Brödel et al. demonstrated in situ gene editing of gut bacteria using phage-derived particles to enhance gut barrier function and reduce inflammation. These advances point toward a new era of personalized microbiome-based therapies that directly address the underlying mechanisms of gut permeability-related disorders.

## 8. Conclusions and Future Directions

This review highlights the growing recognition of intestinal barrier dysfunction and microbiota dysbiosis as central contributors to Parkinson’s disease (PD) pathogenesis. Disruption of tight junctions and degradation of the mucus layer—driven by pro-inflammatory cytokines such as TNF-α and IL-1β—compromises epithelial integrity, allowing microbial products, like lipopolysaccharide (LPS), to enter the systemic circulation. This, in turn, drives chronic inflammation, promotes α-synuclein aggregation, and accelerates dopaminergic neurodegeneration through the gut–brain axis. Microbial shifts further exacerbate this cycle. Reductions in beneficial taxa, such as *Faecalibacterium prausnitzii*, *Lachnospiraceae*, and Bifidobacterium breve, alongside altered levels of *Akkermansia muciniphila*, may weaken barrier function and reduce anti-inflammatory signaling. Meanwhile, the increased abundance of pro-inflammatory genera like certain Bacteroidetes and facultative anaerobes has been associated with greater permeability and immune activation. Yet, the role of specific taxa remains inconsistent across studies, and the precise microbial drivers of gut dysfunction in PD are still unclear.

A key unanswered question is which microbial taxa and functional metabolites are most protective of gut barrier integrity in PD and how their loss contributes to disease onset and progression. To address this, several future directions should be pursued. Longitudinal studies incorporating microbiome sequencing and untargeted metabolomics across disease stages are essential for identifying consistent microbial and metabolic alterations. Mechanistic studies will be essential for understanding how microbial-derived metabolites, especially SCFAs, and the effects of immune mediators influence intestinal permeability, α-synuclein pathology, and neuroinflammation. These insights should inform human clinical trials aimed at evaluating whether targeted dietary interventions, probiotic supplementation, or microbial metabolite restoration can improve gut barrier function and PD symptomatology.

Equally important is the integration of genetic stratification into therapeutic design. Understanding how specific genetic variants modulate responses to microbiota-based therapies may allow for better identification of patient subgroups who are most likely to benefit from specific interventions, including regenerative strategies. Taken together, these efforts may facilitate a shift from symptomatic treatment to a mechanism-based, individualized strategy that targets both peripheral and central drivers of Parkinson’s disease.

## Figures and Tables

**Table 1:** Current and Emerging Therapies Targeting Gut Permeability in PD.

Category	Subcategory	Treatment	Mechanism
Traditional PD Medications	Dopaminergic Medications	Levodopa/Carbidopa	Alleviates motor symptoms but may disrupt gut motility
		Dopamine agonists (e.g., pramipexole, ropinirole)	Impact gut function through the enteric nervous system
	Enzyme Inhibitors	MAO-B inhibitors (e.g., selegiline, rasagiline)	Potential neuroprotective effects extend to the enteric nervous system
		COMT inhibitors (e.g., entacapone)	May influence the gut metabolism of levodopa
	Anticholinergics		Used for tremor control, can affect gut motility and potentially influence intestinal permeability
Gut-Specific Interventions	Microbiome-Targeted Interventions	Probiotics—*Akkermansia muciniphila*, *Bifidobacterium*	Enhances mucus production, reduces inflammation
		Prebiotics—Galactooligosaccharides	Stimulates *Bifidobacterium* growth, improves barrier function
		Fecal microbiota transplantation (FMT)—Healthy donor microbiota	Restores microbial diversity and reduces α-syn burden
		SCFA-boosting diets	Restore microbial diversity and reinforce barrier function via GPR41/43 signaling
	Anti-inflammatory Agents		To reduce gut inflammation and potentially improve barrier function
	Immunomodulators		To regulate immune responses and mitigate chronic inflammation in the gut
Emerging Therapies	Mucus Layer Enhancers		Therapies targeting MUC2 production to strengthen the protective mucus barrier
	Tight Junction Modulators	Larazotide acetate	Inhibits zonulin and stabilizes tight junction integrity (e.g., targeting occludin, claudins, ZO-1)
	Personalized Microbiome Engineering	Phage-delivered gene editing	Tailored approaches to enhance specific beneficial bacterial populations, and enhance SCFA producers
	Neuroprotective Agents		Compounds that may benefit both gut and brain health

## Data Availability

No new data were created or analyzed in this study. Data sharing is not applicable to this article.

## Abbreviations

The following abbreviations are used in this manuscript:

PD	Parkinson’s disease
BBB	Blood–brain barrier
IBD	Inflammatory bowel disease
LPS	Lipopolysaccharides
SCFAs	Short-chain fatty acids
CNS	Central Nervous System
